# Microwave-Assisted Hydrothermal Rapid Synthesis of Ultralong Hydroxyapatite Nanowires Using Adenosine 5′-Triphosphate

**DOI:** 10.3390/molecules27155020

**Published:** 2022-08-07

**Authors:** Yu Zhang, Ying-Jie Zhu, Han-Ping Yu

**Affiliations:** 1 College of Chemistry and Materials Science, Shanghai Normal University, Shanghai 200234, China; 2 State Key Laboratory of High Performance Ceramics and Superfine Microstructure, Shanghai Institute of Ceramics, Chinese Academy of Sciences, Shanghai 200050, China; 3 Center of Materials Science and Optoelectronics Engineering, University of Chinese Academy of Sciences, Beijing 100049, China

**Keywords:** nanowire, hydroxyapatite, microwave, hydrothermal, adenosine triphosphate

## Abstract

Ultralong hydroxyapatite (HAP) nanowires are promising for various biomedical applications owing to their chemical similarity to the inorganic constituent of bone, high biocompatibility, good flexibility, excellent mechanical properties, etc. However, it is still challenging to control the formation of ultralong HAP nanowires because of the presence of free PO_4_^3^^−^ ions in the reaction system containing the inorganic phosphate source. In addition, it takes a long period of time (usually tens of hours) for the synthetic process of ultralong HAP nanowires. Herein, for the first time, we have developed an eco-friendly calcium oleate precursor microwave hydrothermal method using biocompatible adenosine 5′-triphosphate (ATP) as a bio-phosphorus source and water as the only solvent for the rapid synthesis of ultralong HAP nanowires. The controllable hydrolysis of ATP can avoid the premature formation of calcium phosphate nuclei and uncontrollable crystal growth. Microwave heating can significantly shorten the synthetic time from tens of hours required by the traditional heating to 1 h, thus achieving high efficiency, energy saving and low cost. The as-prepared ultralong HAP nanowires with high flexibility have lengths of several hundred micrometers and diameters of 10~20 nm, and they usually self-assemble into nanowire bundles along their longitudinal direction. The as-prepared ultralong HAP nanowire/chitosan porous scaffold has excellent bioactivity, good biodegradation and cytocompatibility owing to the bioactive adenosine adsorbed on the surface of ultralong HAP nanowires. It is expected that ultralong HAP nanowires will be promising for various applications in the biomedical fields, such as bone defect repair, skin wound healing, and as a drug nanocarrier.

## 1. Introduction

Hydroxyapatite (HAP, Ca_10_(PO_4_)_6_(OH)_2_) is the main inorganic constituent of bone. HAP biomaterials have attracted much attention owing to their chemical similarity to the inorganic component of bone, high biocompatibility, good biodegradation ability, excellent osteoinductivity and osteoconductivity [1,2]. The degradation products of HAP (i.e., Ca^2+^ and PO_4_^3^^−^ ions) are nontoxic and absorbable in vivo, which is favorable for bone regeneration [3]. Previous studies have also verified that HAP biomaterials are ideal for bone grafting, which can promote the bone regeneration and angiogenesis to accelerate the process of bone defect repair [4]. HAP biomaterials have been applied clinically in orthopedic and dental repair [3,5]. In addition, HAP biomaterials have also been applied in other biomedical areas, such as drug delivery [6,7,8,9,10,11,12,13], bioactive coating [14,15], dental materials [16,17], and antitumor therapy [3,18].

The properties of HAP biomaterials are determined by their crystal structure, morphology and size [19]. Among various morphologies of HAP structures, HAP nanowires, whose lengths are significantly larger than diameters, are of great interest due to their structural superiority and excellent mechanical properties. HAP nanowires show a similar morphology to HAP nanostructures in human hard tissues. For example, the tooth enamel is formed by the self-assembly of aligned HAP nanorods [20]. Needle-shaped HAP crystals are considered as assembly building blocks of the bone [21]. Moreover, the porous scaffold made of HAP nanowires can mimic the in vivo environment for cells.

Recently, the authors’ research group put forward the term “ultralong hydroxyapatite nanowires”. The ultralong hydroxyapatite nanowires are defined as the hydroxyapatite nanowires with diameters smaller than 100 nm, lengths larger than 100 μm, and aspect ratios higher than 1000 [22,23,24]. Furthermore, the authors’ research group developed the calcium oleate precursor solvothermal method for the synthesis of ultralong hydroxyapatite nanowires with diameters of 10~20 nm and lengths up to several hundred micrometers [25,26,27,28,29]. In the calcium oleate precursor solvothermal method, ultralong HAP nanowires are synthesized typically using CaCl_2_, oleic acid, NaH_2_PO_4_·2H_2_O, NaOH, water, and ethanol (or methanol). The calcium oleate precursor solvothermal method could be extended to the synthesis of ultralong HAP nanowires using various monohydroxy alcohols [26] and different phosphate salts [27]. As an example, ultralong HAP nanowires with lengths of close to 1000 μm could be prepared by the calcium oleate precursor solvothermal method using methanol instead of ethanol [26]. The aspect ratios of ultralong HAP nanowires could reach as high as >10,000, leading to high flexibility, which can solve the problems of high brittleness and hardness of the traditional HAP materials [28,29]. On the other hand, ultralong HAP nanowires, from the structural standpoint, have advantages of high flexibility, high toughness and high strength, and can be interwoven into a high-strength porous networked structure [22,23]. Generally, the higher aspect ratios of HAP nanowires (with nanoscale diameters and larger lengths) can greatly enhance their properties and thus promote their diverse applications [29,30].

However, in the calcium oleate precursor solvothermal method, organic solvents are adopted for the synthesis of ultralong HAP nanowires. Later, the authors’ group developed a more eco-friendly and low-cost calcium oleate precursor hydrothermal method for the synthesis of ultralong HAP nanowires using water as the only solvent without any organic solvent [31]. In addition, the highly efficient calcium oleate precursor microwave hydrothermal method was also developed for the rapid and energy-saving synthesis of ultralong HAP nanowires [32]. Furthermore, ultralong HAP nanowires have been adopted as the building material to prepare the flexible inorganic paper sheets with various functions, which are promising for a variety of applications, such as the fire resistance [22,23,25], fire-retardant high-temperature-resistant label paper and Xuan paper [33,34], waterproof [35], water purification [36,37], multimode anti-counterfeiting [38], information encryption and decryption [39], solar energy-driven desalination of seawater [40], and biomedical applications [41,42,43,44]. In particular, the ultralong HAP nanowire-based biopapers, hydrogels, aerogels, and bone repair scaffolds are highly biocompatible and bioactive to promote the new bone growth inside the porous network in vivo, providing a series of potential clinical alternatives for bone defect repair and skin wound healing [41,42,43,44,45,46].

The formation of HAP nanostructures undergoes intricate processes, during which calcium phosphate intermediate phases form depending on the experimental factors such as concentrations of reactants, pH value, reaction time and temperature [47]. The inorganic phosphate salts as the phosphorus source would rapidly form free phosphate ions and react with calcium ions in aqueous solution, forming calcium phosphate nuclei prematurely, leading to the difficulty in controlling the nucleation and crystal growth. Additionally, the osteoinductive properties of the HAP scaffolds could be enhanced by designing their hierarchical porous structure similar to bone [48], but are still lower than those containing bioactive components (such as bone morphogenetic proteins, collagen and osteonectin). It was found that the porous scaffold consisting of only ultralong HAP nanowires showed a lower rate to induce new bone formation than those of ultralong HAP nanowire-based composite scaffolds containing bioactive components, such as magnesium silicate and collagen [49,50], which can provide many binding sites for the cells and can directly mediate the cell behaviors and fate.

Adenosine 5′-triphosphate (ATP) is the direct energy source in the living body and plays an important role in life activities [51,52]. An ATP molecule contains one adenosine (which made of an adenine and a ribose) and three phosphate groups [53]. The bioactive adenosine group from the ATP molecule can promote the osteogenic differentiation of human bone marrow mesenchymal stem cells to accelerate new bone formation [54]. ATP is also an excellent bio-phosphorus source for the preparation of calcium phosphate-based biomaterials [53,55,56]. One of the advantages is that the bioactive ATP molecules and adenosine groups can be in situ decorated on the surface of HAP biomaterials with high bioactivity. On the other hand, the hydrolysis of ATP molecules highly depends on specific experimental conditions. Calcium phosphate nanomaterials with various hierarchical structures were synthesized by changing the hydrolysis environment of ATP [57,58,59,60]. But, unfortunately, most products synthesized by the above methods exhibit the morphologies of nanoparticles, nanorods and short nanowires, and these nanostructures make it difficult to construct stable porous networked structures.

In this work, using ATP as the bio-phosphorus source, ultralong HAP nanowires are rapidly synthesized by the calcium oleate precursor microwave hydrothermal method using water as the only solvent without any organic solvent. ATP cannot hydrolyze in aqueous solution at room temperature, avoiding the premature reaction between calcium ions and phosphate ions. The microwave heating is applied to rapidly raise the temperature of the reaction system. As a result, the as-prepared ultralong HAP nanowires have relatively uniform lengths and small diameters, and they assemble in an orderly manner along their longitudinal direction into nanowire bundles at the nanoscale with a high flexibility. Inspired by the structure of cancellous bone, ultralong HAP nanowires are used to construct the hierarchically porous scaffold with a stable porous networked structure, high porosity and suitable biodegradation ability. Owing to the bioactive adenosine adsorbed on the surface of ultralong HAP nanowires, the as-prepared hierarchically porous scaffold possesses excellent bioactivity and biocompatibility, which is promising for various biomedical applications such as bone defect repair.

## 2. Experimental

### 2.1. Materials and Chemicals

Calcium chloride (CaCl_2_), sodium dihydrogen phosphate dihydrate (NaH_2_PO_4_·2H_2_O) and acetic acid were purchased from Sinopharm Chemical Reagent Co., Ltd. (Shanghai, China). Sodium oleate and chitosan were purchased from Aladdin Industrial Corporation. Adenosine 5′-triphosphate disodium salt (Na_2_ATP) and adenosine 5′-monophosphate disodium salt (Na_2_AMP) were obtained from Sigma-Aldrich. Ethanol, ethylene glycol, and methanol were obtained from Shanghai Lingfeng Chemical Reagent Co., Ltd. (Shanghai, China). All chemicals were used as received without further purification. Deionized water was used in the related experiments.

### 2.2. Synthesis of Ultralong HAP Nanowires

Ultralong HAP nanowires were synthesized by the calcium oleate precursor microwave hydrothermal method. Briefly, sodium oleate (2.634 g) was dissolved in 15 mL of deionized water. Next, CaCl_2_ (0.110 g) aqueous solution (5 mL) was added into the above sodium oleate aqueous solution under magnetic stirring at room temperature. After 15 min stirring, 5 mL aqueous solution containing 0.367 g Na_2_ATP was added dropwise to the above mixture under magnetic stirring at room temperature. The resulting suspension was transferred into an autoclave, sealed and heated in a microwave oven at 180 °C for 60 min. The autoclave was a modified polytetrauoroethylene (TFM) cylindrical autoclave (volume 60 mL, inner diameter 3.0 cm, outer diameter 3.8 cm, and inner height 9.3 cm), and it was placed in a high-strength outer vessel. The microwave oven (MDS-6, Sineo, Shanghai, China, frequency 2.45 GHz, maximum power 1000 W) was a microwave-hydrothermal synthesis system with a continuous heating mode. Temperature was measured with a platinum resistor temperature sensor (temperature range 0–250 °C) and controlled by automatic adjusting of the microwave power. The autoclave was rotating continuously during microwave irradiation for relatively uniform heating. After cooling to room temperature, the product was washed with ethanol and deionized water three times, respectively, and then dried at 60 °C for 24 h. Other samples were prepared by similar procedures but varying experimental parameters. Ultralong HAP nanowires were also prepared using Na_2_AMP (0.783 g) as the phosphorus source by similar procedures but replacing Na_2_ATP with Na_2_AMP.

The calcium oleate precursor microwave solvothermal method was also extended to the rapid synthesis of ultralong HAP nanowires in a mixed solvent reaction system. 4.5 mL of deionized water, 3.5 mL of oleic acid and 2 mL of methanol (or ethanol) were mixed uniformly. After that, 5 mL aqueous solution of NaOH (0.350 g), 5 mL aqueous solution of CaCl_2_ (0.110 g) and 5 mL aqueous solution of ATP (0.367 g) were added dropwise, respectively. The resulting suspension was transferred into a 60 mL autoclave, sealed and heated in a microwave oven (MDS-6, Sineo, Shanghai, China) at 180 °C for 60 min. After cooling to room temperature, the product was washed with ethanol and deionized water three times, respectively.

To analyze the reaction mechanism between the calcium oleate precursor and Na_2_ATP, the calcium oleate precursor (reaction product without adding the phosphorus source), and the reaction product between calcium oleate and inorganic phosphate (NaH_2_PO_4_·2H_2_O) were used for comparison. Specifically, an aqueous solution (5 mL) containing 0.110 g CaCl_2_ was added into an aqueous solution (15 mL) containing 2.634 g sodium oleate under magnetic stirring for 15 min to form the calcium oleate precursor, then the precursor was washed with methanol and methanol-ethylene glycol (1:1, vol.%) thrice, respectively, and dried in vacuum at room temperature. For the preparation of the reaction product between calcium oleate and the phosphorus source, the above calcium oleate precursor aqueous suspension was further mixed with an aqueous solution (5 mL) containing 0.367 g Na_2_ATP, and an aqueous solution (5 mL) containing 0.312 g NaH_2_PO_4_·2H_2_O for 15 min, respectively, following the same washing and drying procedures.

### 2.3. Preparation of the Ultralong HAP Nanowire/Chitosan (CS) Scaffold

3.000 g CS was added to 97.000 g of acetic acid aqueous solution (1 vol.%) under stirring in a water bath at 60 °C. Ultralong HAP nanowires were then mixed with the above CS solution with a HAP/CS weight ratio of 7:3 under stirring for 15 min at room temperature. After that, the resulting suspension was transferred to the 96-well plate, and freeze-dried.

### 2.4. Characterization

X-ray powder diffraction (XRD) patterns were recorded using an X-ray diffractometer (Rigaku D/max 2550 V, Cu K_α_ radiation, λ = 1.54178 Å). Fourier transform infrared (FTIR) spectra were obtained on a FTIR spectrometer (FTIR-7600, Lambda Scientific, Australia). X-ray photoelectron spectroscopy (XPS) patterns were measured on an X-ray photoelectron spectrometer (Thermo Fisher Scientific, Waltham, MA, USA). Scanning electron microscopy (SEM) images were obtained using scanning electron microscopes (Hitachi S-4800 and TM-3000, Tokyo, Japan). Transmission electron microscopy (TEM) images were obtained using a transmission electron microscope (JEM-2100F, JEOL, Tokyo, Japan). The thermal properties of the samples were tested by the thermogravimetric (TG) analysis and differential scanning calorimetry (DSC) (STA 409/PC, Netzsch, Selb, Germany) at a heating rate of 10 °C·min^−1^ in flowing air.

The compressive strength and strain of the samples were tested using a universal testing machine (DRK 101A, Drick, Jinan, China) at room temperature under a relative humidity of about 45%, and the testing specimens had a cylinder shape with a diameter of 15 mm and a height of 15 mm. The Brunauer–Emmett–Teller (BET) specific surface areas of the samples were measured by a surface area and pore size analyzer (Tristar II 3020, Micromeritics, Norcross, GA, USA). The porosity of the sample was measured by immersing the sample in ethanol for 1 h, and calculated using the following equation:

Porosity (%) = (Δ*m*/*ρ*)/*V*_0_ × 100%

where Δ*m* is the mass difference of the sample after and before the absorption of ethanol, *ρ* is the density of ethanol, and *V*_0_ is the apparent volume of the sample.

### 2.5. Ion Release from the Ultralong HAP Nanowire Scaffold

The ultralong HAP nanowire scaffold (40 mg) was immersed in the normal saline (30 mL) at 37 °C under a constant shaking rate (160 rpm) in a desk-type constant-temperature oscillator (HZQ-X 160, Suzhou, China). 1 mL of the supernatant was withdrawn and replaced with 1 mL fresh normal saline at certain time points. The supernatant was diluted by 3% nitric acid and measured by inductively coupled plasma-optical emission spectrometry (JY 2000-2, Horiba, Paris, France) to determine the concentrations of Ca^2+^ ions.

### 2.6. Mineralization Tests

The apatite-forming ability of the as-prepared HAP nanowire/CS scaffold was tested in 1.5 × simulated body fluid (SBF). The samples (2 mg each) were soaked in 4 mL of SBF at 37 °C for one day, three days and seven days, respectively, then washed with deionized water thrice and freeze-dried. The morphologies of the scaffolds were observed by SEM.

### 2.7. In Vitro Cytotoxicity of the HAP Nanowire/CS Scaffold

A cell Counting Kit-8 assay (CCK-8; Dojindo Molecular Technologies) was used to determine the cytotoxicity of the HAP nanowire/CS scaffold. Briefly, rat bone marrow mesenchymal stem cells (rBMSCs) cells were seeded in a 96-well plate at a density of 2 × 10^3^ cells/well and cultured with leaching solution from the HAP nanowire/CS scaffold (3 cm^2^/mL). At days one, three and five, the culture medium was removed and 100 μL of fresh medium with 10% CCK-8 solution was added to each well. After incubation for 2 h, aliquots (100 μL) from each well were transferred to a new 96-well plate for measurement. The absorbance at a wavelength of 450 nm was measured with a microplate reader (Bio-Rad 680, Hercules, CA, USA).

After seeding for three days, the viability of rBMSCs was further assessed using the Live/Dead Cell Viability Assay Kit (Invitrogen) according to the manufacturer’s instructions, and the rBMSCs were observed using a confocal laser scanning microscope (CLSM; LSM 510, Carl Zeiss, Oberkochen, Germany).

## 3. Results and Discussion

### 3.1. Characterization of Ultralong HAP Nanowires

**Figure 1:**
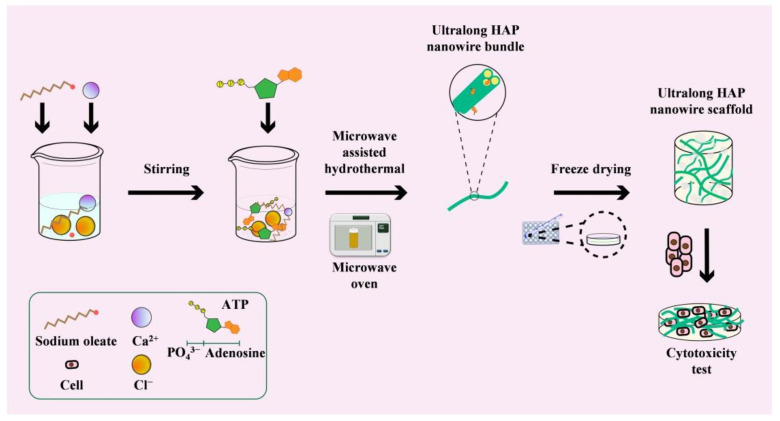
Schematic diagram for the rapid synthesis of ultralong HAP nanowires by the calcium oleate precursor microwave hydrothermal method using ATP as a bio-phosphorus source, and the preparation of the ultralong HAP nanowire porous scaffold.

Ultralong HAP nanowires can be rapidly synthesized using Na_2_ATP as a bio-phosphorus source by the calcium oleate precursor microwave hydrothermal method. The schematic diagram for the rapid synthesis of ultralong HAP nanowires by the calcium oleate precursor microwave hydrothermal method, and the preparation of the HAP nanowire/chitosan porous scaffold is presented in Figure 1. The use of ATP as a bio-phosphorus source has several advantages. For example, there are no free PO_4_^3^^−^ ions in the initial reaction system because the phosphate groups exist in the ATP biomolecules, thus the premature formation of calcium phosphate nuclei and uncontrollable crystal growth can be avoided. The PO_4_^3^^−^ ions are formed by the hydrolysis of ATP molecules in aqueous solution, and it can be controlled by adjusting experimental conditions such as reaction temperature and time. In addition, microwave heating can rapidly raise the reaction temperature and accelerate the processes of chemical reactions, nucleation and crystal growth, thus significantly shortening the synthetic time and achieving high efficiency and saving energy.

**Figure 2:**
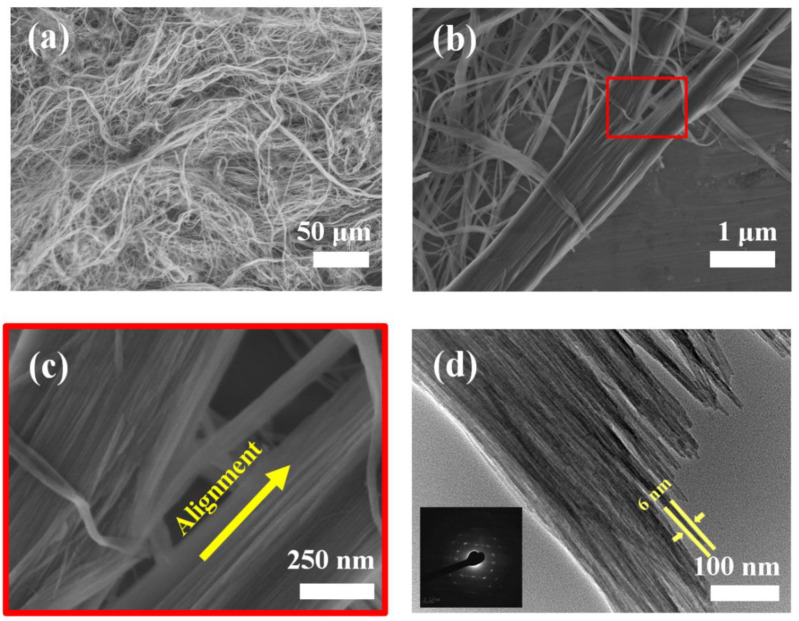
SEM images (**a**–**c**) and TEM image (**d**) of ultralong HAP nanowires synthesized by the calcium oleate precursor microwave hydrothermal method using ATP as a bio-phosphorus source in aqueous solution at 180 °C for 60 min; (**c**) is the enlarged SEM image in the area labelled with a red rectangle in (**b**); the inset of (**d**) is a selected-area electron diffraction pattern of ultralong HAP nanowires.

Figure 2a–c shows SEM images of ultralong HAP nanowires synthesized using ATP as a bio-phosphorus source in aqueous solution by the calcium oleate precursor microwave hydrothermal method. One can see that the as-prepared ultralong HAP nanowires have diameters of <20 nm, and lengths of several hundred micrometers. In many cases, ultralong HAP nanowires self-assemble along their longitudinal direction at the nanoscale to form nanowire bundles with larger diameters. This is also consistent with the TEM observation that each nanowire bundle consists of many highly aligned ultralong HAP nanowires along their longitudinal direction (Figure 2d). The as-prepared ultralong HAP nanowires are highly flexible and can even be bent naturally, as shown in Figure 2a. The ultralong HAP nanowires are crystallized, and the nanowire bundle exhibits the single-crystal structure because of the high orientation of self-assembled ultralong HAP nanowires in the nanowire bundle. Ultralong HAP nanowires are very thin, and the diameter of some ultralong HAP nanowires is only as small as about 6 nm, as shown in Figure 2d.

**Figure 3:**
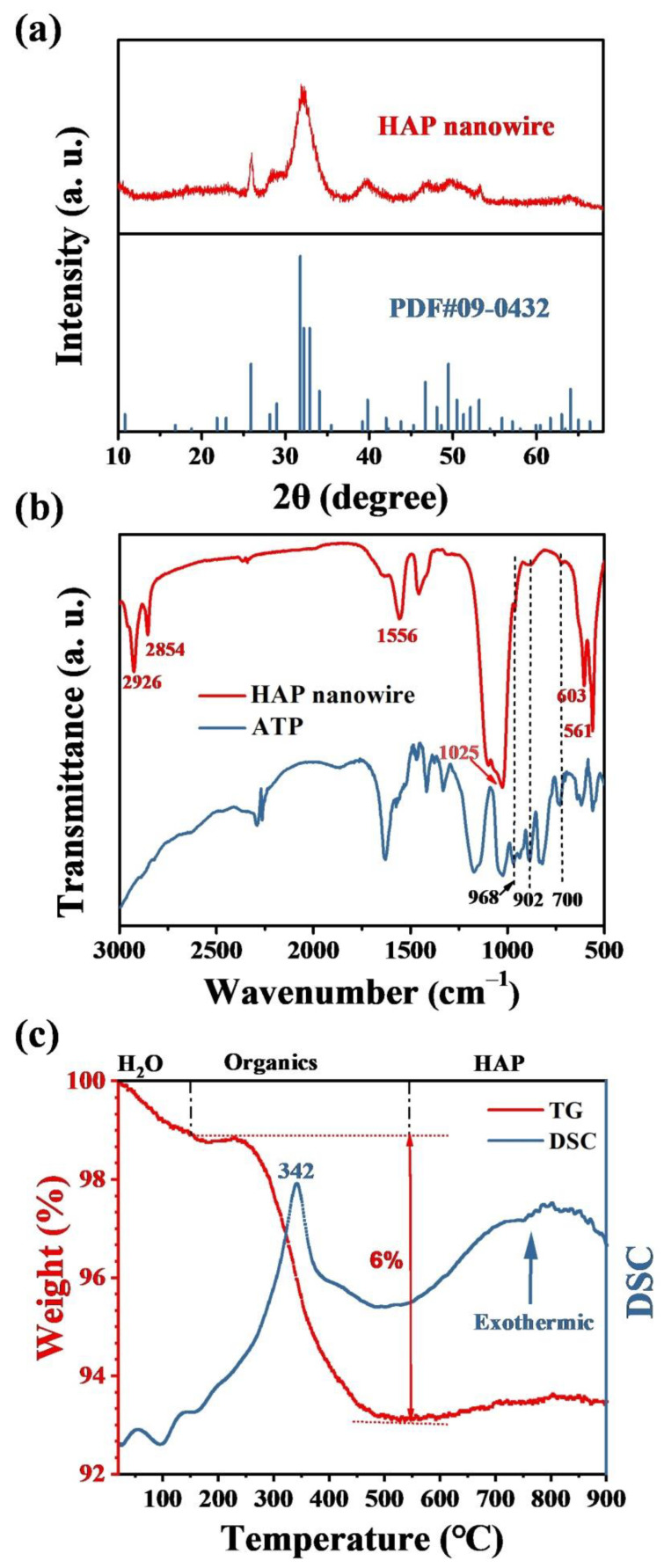
Characterization of ultralong HAP nanowires synthesized by the calcium oleate precursor microwave hydrothermal method using ATP as a bio-phosphorus source in aqueous solution at 180 °C for 60 min. (**a**) XRD pattern. (**b**) FTIR spectra of ultralong HAP nanowires and ATP. (**c**) TG and DSC curves.

The XRD pattern of the as-prepared ultralong HAP nanowires is shown in Figure 3a, which indicates that the as-prepared product is composed of a single crystal phase of hexagonal hydroxyapatite (JCPDS No. 09-0432). The FTIR spectra of ultralong HAP nanowires and Na_2_ATP are shown in Figure 3b. The absorption peaks at 603 and 561 cm^−1^ correspond to the bending mode of the O-P-O of the PO_4_^3−^ group of HAP nanowires. The characteristic absorption peaks at about 2926 and 2854 cm^−1^ are attributed to -CH_3_ and -CH_2_ groups. In addition, the absorption peaks at 968, 902 and 700 cm^−1^ are ascribed to functional groups from ATP, and the absorption peak at 1556 cm^−1^ corresponds to the N-H bond of ATP, indicating the existence of the adenosine group on the surface of ultralong HAP nanowires. The thermal stability of the as-prepared ultralong HAP nanowires is tested by TG and DSC, as shown in Figure 3c. The weight loss of the sample is about 1 wt.% from room temperature to about 150 °C, which is ascribed to the loss of the adsorbed water in the sample. The weight loss of the sample is about 6 wt.% between about 150 and 500 °C, which is attributed to the oxidation and decomposition of the adsorbed adenosine group on the surface of ultralong HAP nanowires, and this weight loss in the TG curve corresponds to an obvious exothermic peak at around 342 °C in the DSC curve.

**Figure 4:**
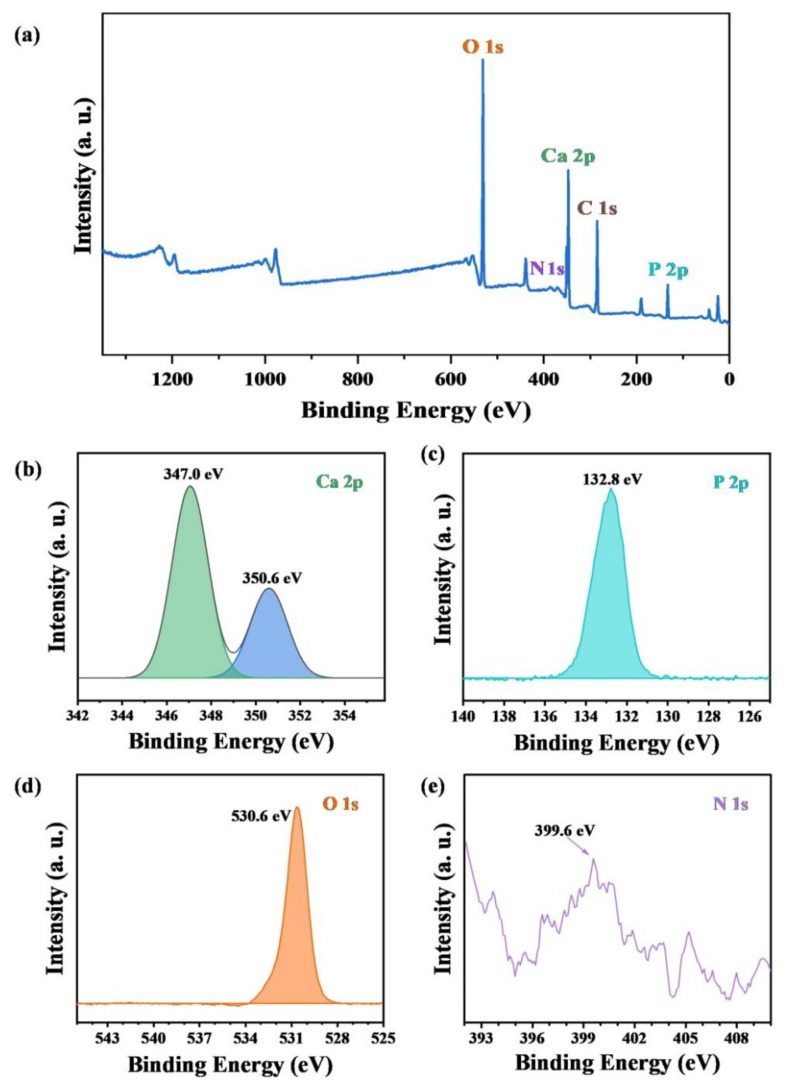
XPS patterns of ultralong HAP nanowires synthesized by the calcium oleate precursor microwave hydrothermal method using ATP as a bio-phosphorus source in aqueous solution at 180 °C for 60 min. (**a**) Survey XPS pattern. (**b**) Ca 2p XPS pattern. (**c**) P 2p XPS pattern. (**d**) O 1s XPS pattern. (**e**) N 1s XPS pattern.

The XPS patterns of the survey, Ca 2p, P 2p, O 1s and N 1s of ultralong HAP nanowires are shown in Figure 4, which confirm the chemical composition of ultralong HAP nanowires. Furthermore, the N 1s signal is detected in the sample, indicating the presence of adenosine groups adsorbed on the surface of ultralong HAP nanowires. As shown in Figure 4e, the N1s XPS peak located at about 399.6 eV is observed. Owing to the high bioactivity of adenosine for bone regeneration, the adsorbed adenosine on the surface of ultralong HAP nanowires can endow the as-prepared ultralong HAP nanowires with a high affinity for cell adhesion and proliferation.

### 3.2. The Effects of Experimental Parameters on the Formation of Ultralong HAP Nanowires

**Figure 5:**
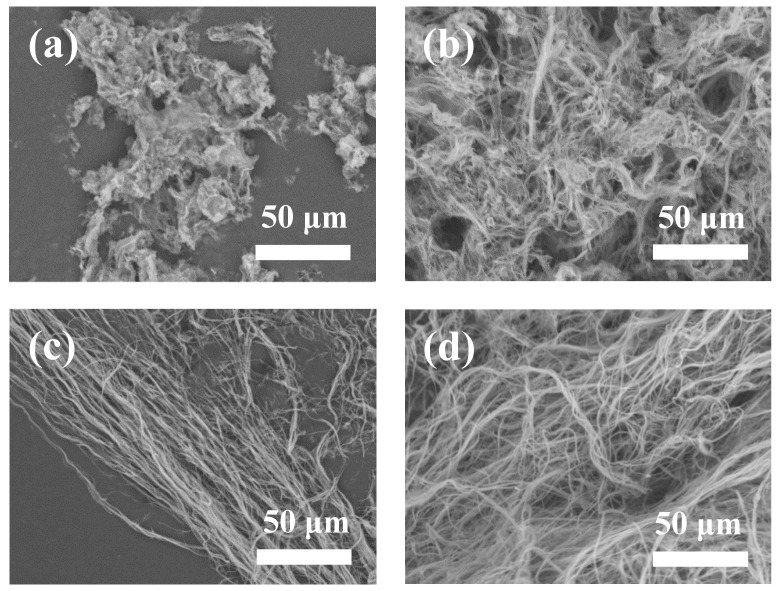
SEM images of the products synthesized using ATP as a bio-phosphorus source in aqueous solution by the calcium oleate precursor microwave hydrothermal method at 180 °C for different times: (**a**) 10 min; (**b**) 20 min; (**c**) 30 min; (**d**) 60 min.

We further investigated the effects of experimental parameters on the final product. Figure 5 shows SEM images of the products synthesized using ATP as a bio-phosphorus source in aqueous solution by the calcium oleate precursor microwave hydrothermal method at 180 °C for different times. As shown in Figure 5a, the products consist of particles as the main product and nanowires as the minor product when the microwave hydrothermal time is 10 min at 180 °C. Ultralong HAP nanowires are obtained as the microwave hydrothermal time is 20 min at 180 °C (Figure 5b). When the microwave hydrothermal time increases to 30 and 60 min at 180 °C, well-defined ultralong HAP nanowires with lengths of several hundred micrometers are formed (Figure 5c,d). That is to say, ultralong HAP nanowires can be rapidly synthesized within 60 min by the calcium oleate precursor microwave hydrothermal method owing to the highly efficient heating way of the microwaves. In contrast, it takes many hours (usually ≥24 h) for the synthesis of ultralong HAP nanowires by the calcium oleate precursor hydrothermal method using the traditional heating approach [31].

**Figure 6:**
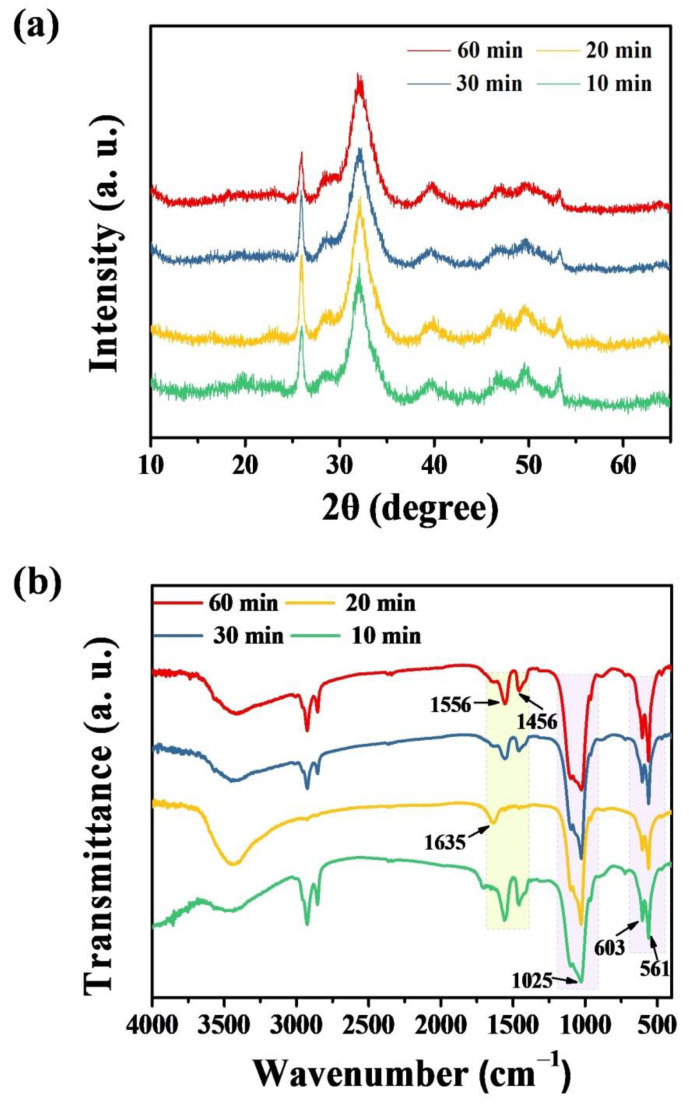
XRD patterns (**a**) and FTIR spectra (**b**) of products prepared using ATP as a bio-phosphorus source in aqueous solution by the calcium oleate precursor microwave hydrothermal method at 180 °C for different times.

Figure 6 shows XRD patterns and FTIR spectra of products prepared using ATP as a bio-phosphorus source in aqueous solution by the calcium oleate precursor microwave hydrothermal method at 180 °C for different times. The XRD results in Figure 6a indicate that the crystalline phase of hydroxyapatite forms in a short period of microwave hydrothermal time (10 min), confirming the high efficiency of the microwave hydrothermal synthesis, leading to the significant saving of both time and energy. The crystalline phase of the product remains hydroxyapatite when the microwave hydrothermal time increases to 20, 30, and 60 min, respectively. In addition, the FTIR spectra of the products prepared by the calcium oleate precursor microwave hydrothermal method at 180 °C for different times further confirm the formation of hydroxyapatite (Figure 6b).

**Figure 7:**
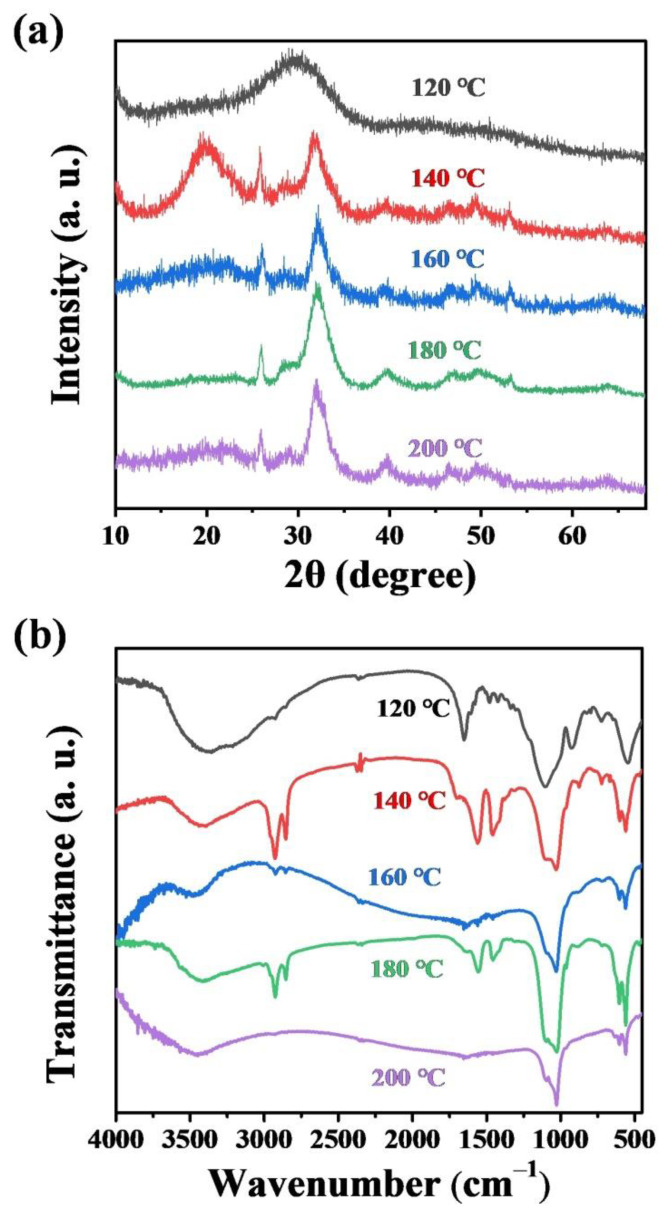
XRD patterns (**a**) and FTIR spectra (**b**) of the products prepared using ATP as a bio-phosphorus source in aqueous solution by the calcium oleate precursor microwave hydrothermal method for 60 min at different temperatures.

In addition, the effect of the microwave hydrothermal temperature on the product was investigated. Figure 7a shows XRD patterns of the products synthesized using ATP as a bio-phosphorus source in aqueous solution by the calcium oleate precursor microwave hydrothermal method for 60 min at different temperatures. The XRD analysis results indicate that sufficiently high reaction temperature is necessary for the formation of ultralong HAP nanowires by the calcium oleate precursor microwave hydrothermal method. The XRD pattern of the product synthesized at 120 °C for 60 min shows a broad peak, corresponding to amorphous calcium phosphate. When the microwave hydrothermal temperature is 140 °C, the crystalline phase of hydroxyapatite appears, but there is still an amorphous phase, evidenced by a broad peak around 2*θ* ≈ 20° in the XRD pattern. When the microwave hydrothermal temperature is higher than 160 °C, a single crystal phase of hydroxyapatite can be synthesized (Figure 7a). Ultralong HAP nanowires form at the reaction temperature of 180 °C and higher than 180 °C (Figure S1 in the Supplementary Materials), indicating that sufficiently high reaction temperature is necessary for the synthesis of ultralong HAP nanowires.

The Ca/P molar ratio of the reaction system also affects the morphology of the product. Figure S2 in the Supplementary Materials shows SEM images of the products synthesized using ATP as a bio-phosphorus source in aqueous solution by the calcium oleate precursor microwave hydrothermal method at 180 °C for 60 min with different Ca/P molar ratios. When the Ca/P molar ratio is 0.5 or lower, ultralong HAP nanowires can be synthesized by the calcium oleate precursor microwave hydrothermal method at 180 °C for 60 min (Figure S2a–c in the Supplementary Materials). In contrast, when the Ca/P molar ratio is higher than 0.5, ultralong HAP nanowires are not formed (Figure S2d in the Supplementary Materials).

**Figure 8:**
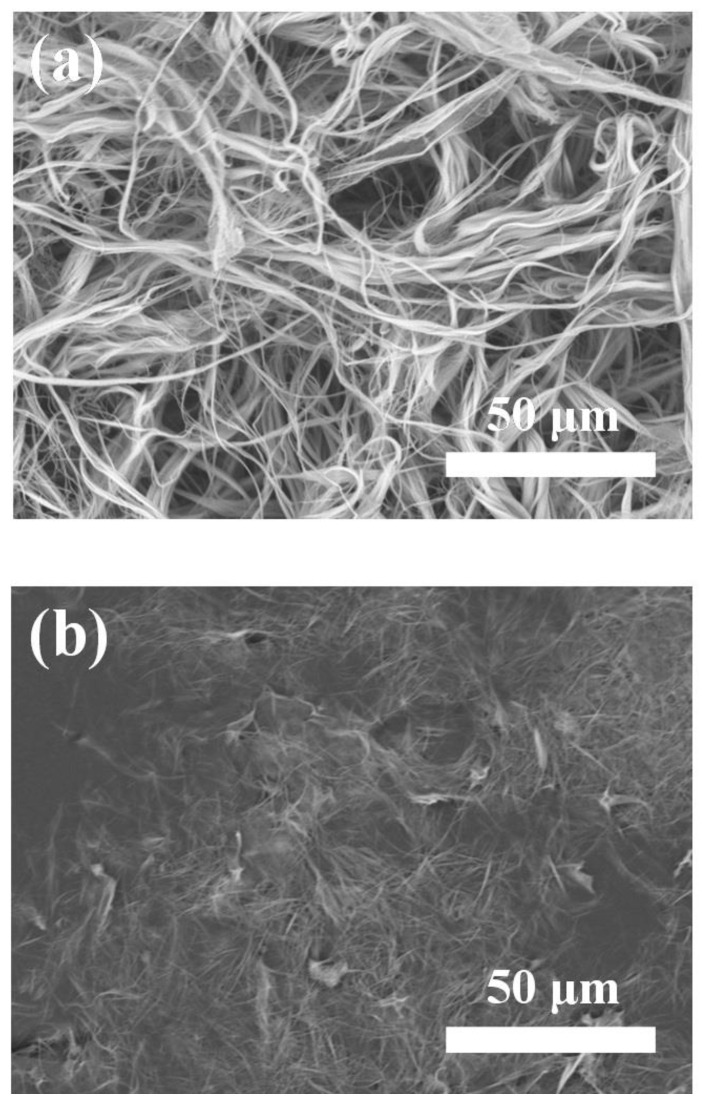
SEM images of the products synthesized using calcium oleate precursor and ATP by the calcium oleate precursor microwave solvothermal method in different reaction systems containing the organic solvent: (**a**) the water/methanol/oleic acid reaction system; (**b**) the water/ethanol/oleic acid reaction system.

As discussed above, ultralong HAP nanowires can be rapidly synthesized by the calcium oleate precursor microwave hydrothermal method using calcium oleate precursor and ATP in aqueous solution. Importantly, this synthetic method can also be extended to the synthesis of ultralong HAP nanowires in the reaction system containing the organic solvent, as shown in Figure 8. The experimental results indicate that ultralong HAP nanowires can be rapidly synthesized using calcium oleate precursor and ATP by the calcium oleate precursor microwave solvothermal method in two reaction systems containing the organic solvent. In addition, ultralong HAP nanowires with much larger lengths can be obtained in the water/methanol/oleic acid reaction system (Figure 8a) than those obtained in the water/ethanol/oleic acid reaction system (Figure 8b). The experimental results indicate that methanol is better than ethanol as the solvent for the synthesis of ultralong HAP nanowires with larger lengths and higher aspect ratios.

### 3.3. The Role of ATP and Formation Mechanism of Ultralong HAP Nanowires

The experimental results indicate that sodium oleate plays an essential role in the formation of ultralong HAP nanowires (Figure S3a,b in the Supplementary Materials). In the absence of sodium oleate, no ultralong HAP nanowires are formed, as shown in Figure S3a in the Supplementary Materials. The product obtained in the absence of sodium oleate consists of calcium hydrogen phosphate (CaHPO_4_) instead of hydroxyapatite, evidenced by the results shown in Figure S3c,d in the Supplementary Materials.

The chemical reactions associated with the formation of HAP nanowires are shown below:

Ca^2+^ + 2R-COO^−^ → Ca(R-COO)_2_ (1)

ATP → Ade + 3PO_4_^3−^ (2)

R-COO^−^ + H_2_O → R-COOH + OH^−^ (3)

10Ca(R-COO)_2_ + 6PO_4_^3−^ + 2OH^−^ → Ca_10_(PO_4_)_6_(OH)_2_ + 20R-COO^−^ (4)

where R = CH_3_(CH_2_)_7_CH=CH(CH_2_)_7_, Ade = adenosine.

Sodium oleate molecules act as a reactant for the chemical reaction with Ca^2+^ ions to form calcium oleate, which acts as the precursor for the formation of ultralong HAP nanowires. In addition, sodium oleate molecules can also act as the surfactant agent in the reaction system, and oleate groups are preferentially adsorbed on the crystal planes along the *a* and *b* axes of HAP crystals, which can inhibit the crystal growth along the *a* and *b* axes, leading to preferential crystal growth along the *c*-axis of hydroxyapatite and the formation of ultralong HAP nanowires. and thus HAP crystals have a tendency to grow along the *c* axis to form HAP nanowires with high respect ratios.

**Figure 9:**
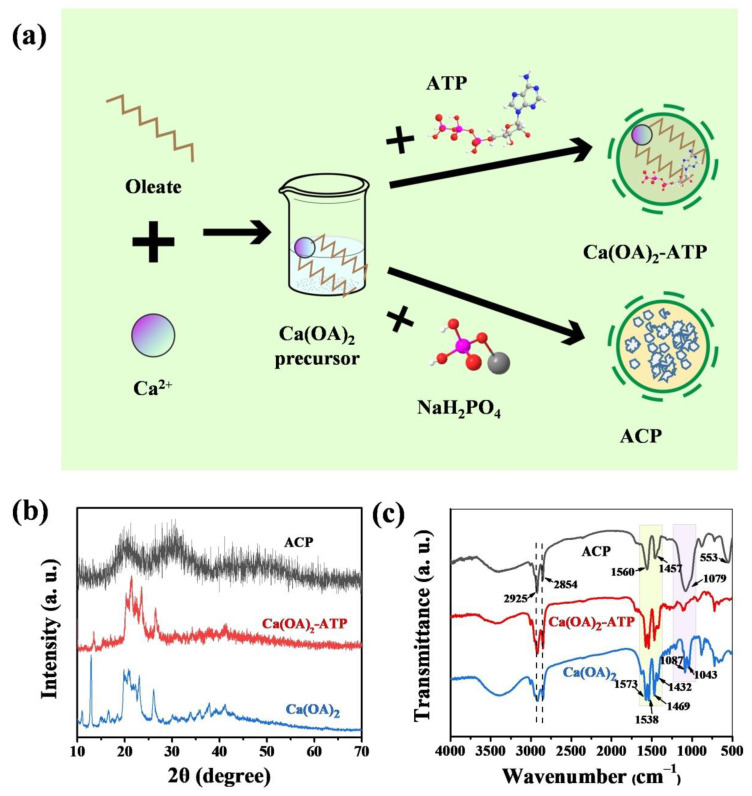
(**a**) Schematic illustration of the initial aqueous reaction system containing Na_2_ATP or NaH_2_PO_4_·2H_2_O as the phosphorus source at room temperature without microwave hydrothermal treatment. (**b**,**c**) XRD patterns (**b**) and FTIR spectra (**c**) of calcium oleate (Ca(OA)_2_) precursor, and the products after mixing calcium oleate precursor with the phosphorus source ATP or NaH_2_PO_4_·2H_2_O in aqueous solution at room temperature.

The controllable hydrolysis of ATP molecules to form free PO_4_^3^^−^ ions in aqueous solution at elevated temperatures, which is different from the inorganic phosphate as the phosphorus source, can avoid the premature chemical reaction between calcium oleate precursor and PO_4_^3^^−^ ions before microwave hydrothermal treatment (Figure 9a). The product obtained after mixing calcium oleate precursor with ATP at room temperature was washed with methanol and methanol-ethylene glycol (1:1, vol.%) to remove the redundant oleate anions and ATP molecules (labelled as Ca(OA)_2_-ATP). For comparison, the control sample was prepared under the same conditions except the use of NaH_2_PO_4_·2H_2_O as the phosphorus source instead of ATP and washed with methanol and methanol-ethylene glycol (1:1, vol.%) (labelled as Ca(OA)_2_-NaH_2_PO_4_). As shown in Figure 9b, the XRD pattern of Ca(OA)_2_-ATP is similar to that of calcium oleate precursor (JCPDS No. 05-0284), indicating that there is no chemical reaction between calcium oleate precursor and ATP at room temperature in aqueous solution. However, the XRD pattern of Ca(OA)_2_-NaH_2_PO_4_ shows two broad and weak diffraction peaks locating around 2*θ* = ~20° and ~30°, respectively, corresponding to the characteristic peaks of amorphous calcium phosphate (ACP). This experimental result indicates that there is a chemical reaction between calcium oleate precursor and free PO_4_^3^^−^ ions to form amorphous calcium phosphate in aqueous solution, and washing the product with methanol and ethylene glycol can inhibit its further transformation to the crystalline phase during the washing step.

The above experimental results are also consistent with the FTIR results. Figure 9c shows the FTIR spectra of the calcium oleate precursor, and the products after mixing the calcium oleate precursor with the phosphorus source ATP or NaH_2_PO_4_·2H_2_O in aqueous solution at room temperature. No obvious difference between Ca(OA)_2_-ATP and calcium oleate precursor in the FTIR spectra is observed. The absorption peaks at 1573, 1538, 1469 cm^−1^ are attributed to the oleate group from calcium oleate, and the absorption peaks at 2925 and 2854 cm^−1^ correspond to the characteristic absorption bands of –CH_3_ and –CH_2_– groups. Whereas the FTIR spectrum of Ca(OA)_2_-NaH_2_PO_4_ shows absorption peaks from the asymmetric stretching vibration of P–O (1079 cm^−1^) and the O–P–O bending vibration (553 cm^−1^) of the PO_4_^3^^−^ group, further confirming the formation of amorphous calcium phosphate [48]. NaH_2_PO_4_ instantly ionizes in aqueous solution at the room temperature, and releases PO_4_^3^^−^, HPO_4_^2^^−^ and H_2_PO_4_^−^ ions, which further react with Ca^2+^ ions from calcium oleate to form amorphous calcium phosphate.

On the contrary, ATP molecules do not hydrolyze in aqueous solution at room temperature so that there are no free PO_4_^3^^–^ ions in the reaction system, avoiding the undesired premature chemical reaction between the calcium oleate precursor and PO_4_^3^^−^ ions. In addition, microwave heating temperature and time can be used to control the hydrolysis rate of ATP molecules, thus further adjusting the chemical reaction rate, nucleation and crystal growth. Moreover, microwave heating can rapidly and uniformly elevate the temperature of the reaction system, leading to high efficiency and the saving of energy and time.

Similarly, ultralong HAP nanowires can also be synthesized by the calcium oleate precursor microwave hydrothermal method using adenosine monophosphate (AMP, also a hydrolysate of ATP) as the phosphorus source. Characterization results including SEM images, XRD pattern and FTIR spectrum of the product synthesized using an aqueous solution containing calcium oleate precursor and AMP by the calcium oleate precursor microwave hydrothermal method at 180 °C for 60 min are shown in Figure S4 in the Supplementary Materials, indicating the formation of ultralong HAP nanowires.

### 3.4. Characterization of the Ultralong HAP Nanowire/CS Scaffold In Vitro

**Figure 10:**
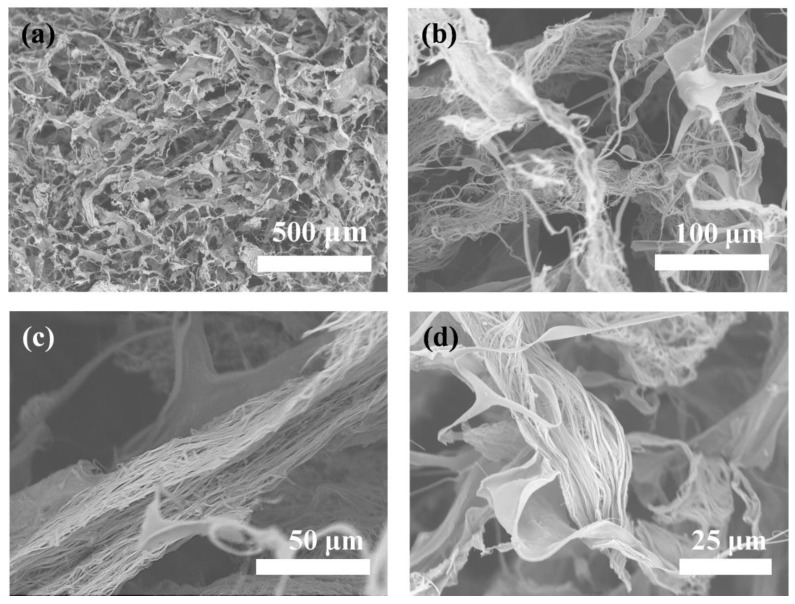
(**a**–**d**) SEM images of the ultralong HAP nanowire/CS porous scaffold.

As discussed above, ATP plays as an important role in the formation of ultralong HAP nanowires. In addition, the hydrolysis product of ATP, i.e., adenosine, endows ultralong HAP nanowires with a high bioactivity. A biocompatible porous scaffold based on ultralong HAP nanowires is prepared. To further improve the mechanical properties of the ultralong HAP nanowire scaffold, chitosan (CS) is introduced as the “glue” in the scaffold. Ultralong HAP nanowires interweave with each other to form an interconnected three-dimensional porous structure in the ultralong HAP nanowire/CS scaffold (Figure 10a–d) with a high porosity of 71.9%. The adjacent ultralong HAP nanowires are tightly stuck together by the CS glue, which can greatly enhance the mechanical properties of the scaffold.

The mechanical properties of the ultralong HAP nanowire/CS scaffold was investigated, and the results are shown in Figure S5 in the Supplementary Materials. The ultralong HAP nanowire/CS scaffold has a Young’s modulus of 10.10 ± 1.30 MPa, which is higher than those of the pure ultralong HAP nanowire scaffold. At a lower strain, the HAP nanowire/CS scaffold can recover to its original state after compression and deformation, exhibiting the elastic properties. However, when the strain is high, the pores of the HAP nanowire/CS scaffold collapse, and the deformation is completely unrecoverable after the compression test.

**Figure 11:**
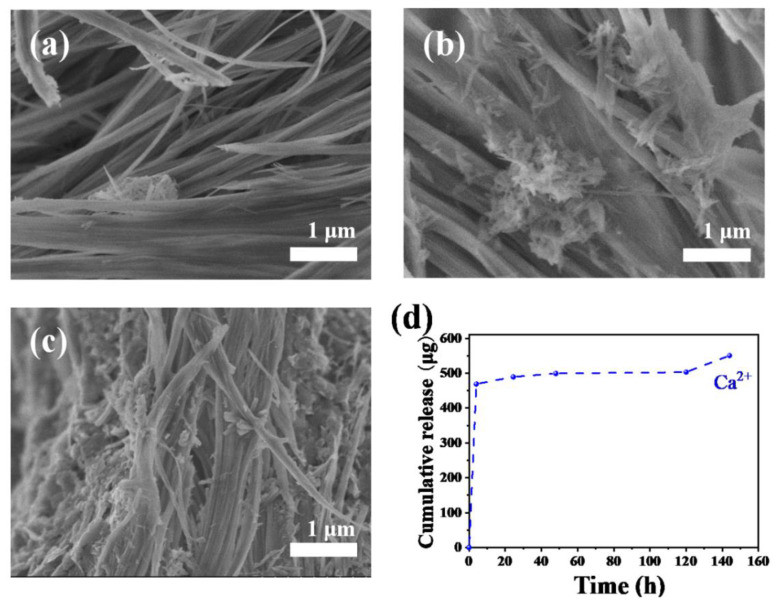
(**a**–**c**) SEM micrographs of the HAP nanowire/CS scaffolds after soaking in 1.5 × SBF for different times: (**a**) one day; (**b**) three days; (**c**) seven days. (**d**) In vitro Ca^2+^ ion release curve of the ultralong HAP nanowire scaffold in normal saline at 37 °C for different times.

To investigate the in vitro osteogenic ability of ultralong HAP nanowires, the HAP nanowire/CS porous scaffolds are immersed in the simulated body fluid (SBF) for different times (one, three, and seven day(s)), respectively, and then observed by SEM. It is found that no significant change is observed on the surface of the HAP nanowire/CS scaffold after immersion in SBF for one day (Figure 11a). Some apatite granules appear on the surface of the HAP nanowire/CS scaffold after immersing in SBF for three days (Figure 11b). More apatite particles are formed on the surface of the HAP nanowire/CS scaffold after immersing in SBF for seven days (Figure 11c), indicating the good bioactivity of the HAP nanowire/CS scaffold.

Moreover, ultralong HAP nanowires can release Ca^2+^ ions in aqueous solution. Figure 11d shows the in vitro Ca^2+^ ion release curve of the ultralong HAP nanowire scaffold in normal saline at 37 °C for different times. The ultralong HAP nanowire scaffold exhibits a sustainable release performance of bioactive ions in normal saline, indicating that the as-prepared ultralong HAP nanowires have good biodegradation ability. Furthermore, the Brunauer–Emmett–Teller (BET) specific surface area of the as-prepared ultralong HAP nanowires is measured to be 44.15 m^2^ g^−1^ (Figure S6 in the Supplementary Materials). The relatively high specific surface area of ultralong HAP nanowires would provide plenty of active sites for cell adhesion, proliferation and differentiation.

**Figure 12:**
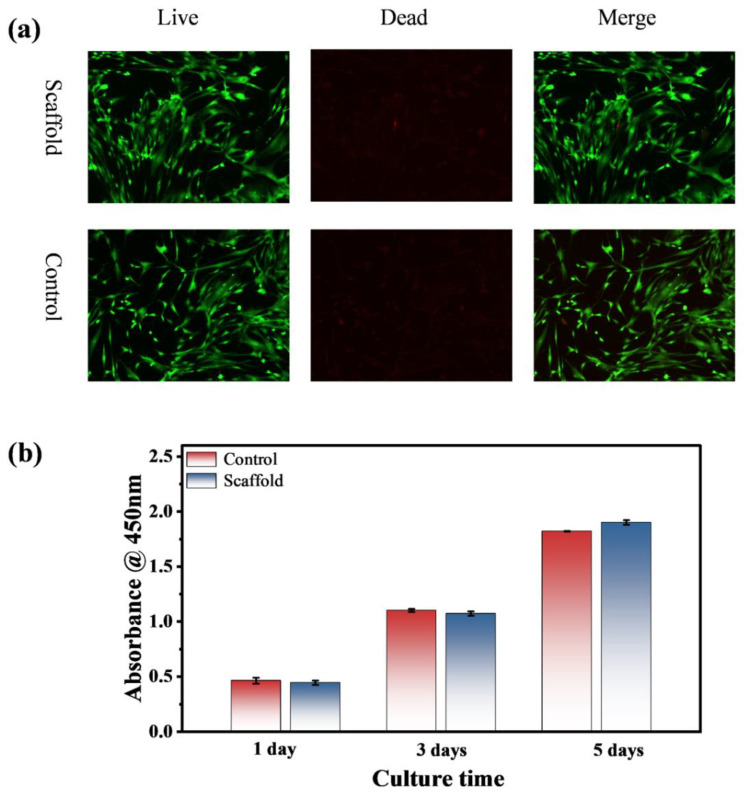
Cell viability assays of the ultralong HAP nanowire/CS scaffold and blank control sample. (**a**) The live/dead staining of rBMSCs cultured with the leaching solution from the ultralong HAP nanowire/CS scaffold after three days (The viable cells are stained green whereas the dead cells are stained red). (**b**) Cell viability of rBMSCs cultured with the ultralong HAP nanowire/CS scaffold for one, three, and five day(s) using the CCK-8 assay.

The cytotoxicity of the ultralong HAP nanowire/CS scaffold was evaluated by the live/dead cell viability assay using rBMSCs. Figure 12 shows cell viabilities of the HAP nanowire/CS scaffold and blank control sample. Figure 12a shows the live/dead staining of rBMSCs cultured with the leaching solution from the HAP nanowire/CS scaffold after three days. The analysis of cytoskeleton staining reveals that the rBMSCs spread well and maintain their phenotype after culture with the leaching solution from the HAP nanowire/CS scaffold after three days, indicating that the as-prepared HAP nanowire/CS scaffold has no toxicity to cells and has good biocompatibility. The CCK-8 assay was performed to evaluate the cell viability of rBMSCs cultured with the HAP nanowire/CS scaffold, as shown in Figure 12b. The HAP nanowire/CS scaffold exhibits no obvious difference in cell viability compared with that of the blank control group within a three day culture, but a little higher cell viability is observed after culture for five days. The experimental results indicate that the released Ca^2+^ and PO_4_^3−^ ions as well as adenosine molecules from the HAP nanowire/CS scaffold are favorable for cell adhesion, spreading and proliferation. In a word, the experimental results prove that the as-prepared ultralong HAP nanowire/CS scaffold is nontoxic and biocompatible to rBMSCs, which is suitable for various biomedical applications such as bone defect repair.

## 4. Conclusions

To sum up, we have developed a calcium oleate precursor microwave hydrothermal method for the rapid synthesis of ultralong HAP nanowires with high flexibility, biocompatibility and bioactivity using ATP as a bio-phosphorus source in aqueous solution. The controllable hydrolysis of ATP biomolecules to release free PO_4_^3^^−^ ions can avoid the premature formation of calcium phosphate nuclei and uncontrollable crystal growth. Compared with the previously reported calcium oleate precursor solvothermal/hydrothermal method in which tens of hours are necessary for the synthesis of ultralong HAP nanowires, the calcium oleate precursor microwave hydrothermal method using ATP as a bio-phosphorus source has several advantages such as high efficiency, rapidness, time saving, energy saving, and environmental friendliness. PO_4_^3^^−^ ions are formed by the hydrolysis of ATP biomolecules in aqueous solution at sufficiently high temperatures under microwave heating, and the hydrolysis of ATP biomolecules can be controlled by adjusting experimental conditions such as microwave heating temperature and time. Furthermore, microwave heating can rapidly raise the reaction temperature and accelerate the processes of chemical reactions, nucleation and crystal growth, thus significantly shortening the synthetic time, achieving high efficiency and saving energy. The as-prepared ultralong HAP nanowires with high flexibility have lengths of several hundred micrometers and diameters of 10~20 nm, and they usually self-assemble into nanowire bundles with larger diameters along their longitudinal direction. This novel method can also be extended to the water/organic solvent reaction system or to the reaction system using AMP instead of ATP as the bio-phosphorus source for the rapid synthesis of ultralong HAP nanowires. Moreover, to investigate the potential for the application in bone tissue engineering, the as-prepared ultralong HAP nanowires are used as the building blocks to construct the bioactive hierarchical porous scaffold with a high porosity (71.9%), high biocompatibility, and good biodegradation ability, which is similar to the structure of natural cancellous bone. Owing to the bioactive adenosine adsorbed on the surface of ultralong HAP nanowires, the as-prepared ultralong HAP nanowire/chitosan porous scaffold possesses excellent bioactivity and cytocompatibility. It is expected that the as-prepared ultralong HAP nanowires obtained by this novel method are promising for various applications in the biomedical fields, such as bone defect repair, skin wound healing, and as a drug nanocarrier.

## Data Availability

The data presented in this study are available on request from the corresponding author.

## Supplementary Materials

The following supporting information can be downloaded at: https://www.mdpi.com/article/10.3390/molecules27155020/s1, Figure S1: SEM images of the products prepared using ATP as a bio-phosphorus source in aqueous solution by the calcium oleate precursor microwave hydrothermal method for 60 min at different temperatures; Figure S2: SEM images of the products synthesized using ATP as a bio-phosphorus source in aqueous solution by the calcium oleate precursor microwave hydrothermal method at 180 °C for 60 min with different Ca/P molar ratios by changing the concentration of ATP; Figure S3: Characterization of the products prepared using an aqueous solution containing CaCl_2_ and ATP by the microwave-assisted hydrothermal method at 180 °C for 60 min at a Ca/P molar ratio of 0.5 using different amounts of sodium oleate; Figure S4: Characterization of the product synthesized using adenosine 5′-monophosphate (AMP) as a bio-phosphorus source in aqueous solution by the calcium oleate precursor microwave hydrothermal method; Figure S5: Compressive stress-strain curve of the ultralong HAP nanowire/CS porous scaffold; Figure S6: Nitrogen adsorption-desorption isotherms of the as-prepared ultralong HAP nanowires synthesized using ATP as a bio-phosphorus source in aqueous solution by the calcium oleate precursor microwave hydrothermal method at 180 °C for 60 min.

## Sample Availability

Samples are available from the authors.
