# Supplementary material for: Microwave-Assisted Hydrothermal Rapid Synthesis of Ultralong Hydroxyapatite Nanowires Using Adenosine 5′-Triphosphate
Source: Molecules. 2022 Aug 7;27(15):5020. doi: 10.3390/molecules27155020 (PMC9370583; doi:10.3390/molecules27155020)
Supplement: Supplementary file 1 [file molecules-27-05020-s001.zip › molecules-1763181-supplementary.pdf]

Electronic Supplementary Information (ESI†)

Microwave-Assisted Hydrothermal Rapid Synthesis of Ultralong Hydroxyapatite Nanowires Using Adenosine 5’-Triphosphate

Yu Zhang [1,2,3], Ying-Jie Zhu [1,2,3,*], Han-Ping Yu [2,*]

[1] *Y. Zhang, Prof. Dr. Y.-J. Zhu*

College of Chemistry and Materials Science, Shanghai Normal University, Shanghai 200234, China

[2] *Y. Zhang, Prof. Dr. Y.-J. Zhu, Dr. H.-P. Yu*

State Key Laboratory of High Performance Ceramics and Superfine Microstructure, Shanghai Institute of Ceramics, Chinese Academy of Sciences, Shanghai 200050, China

[3] *Y. Zhang, Prof. Dr. Y.-J. Zhu*

Center of Materials Science and Optoelectronics Engineering, University of Chinese Academy of Sciences, Beijing 100049, China

** Correspondence: E-mail: y.j.zhu@mail.sic.ac.cn (Y.-J. Zhu)*

yuhanping@mail.sic.ac.cn (H.-P. Yu)

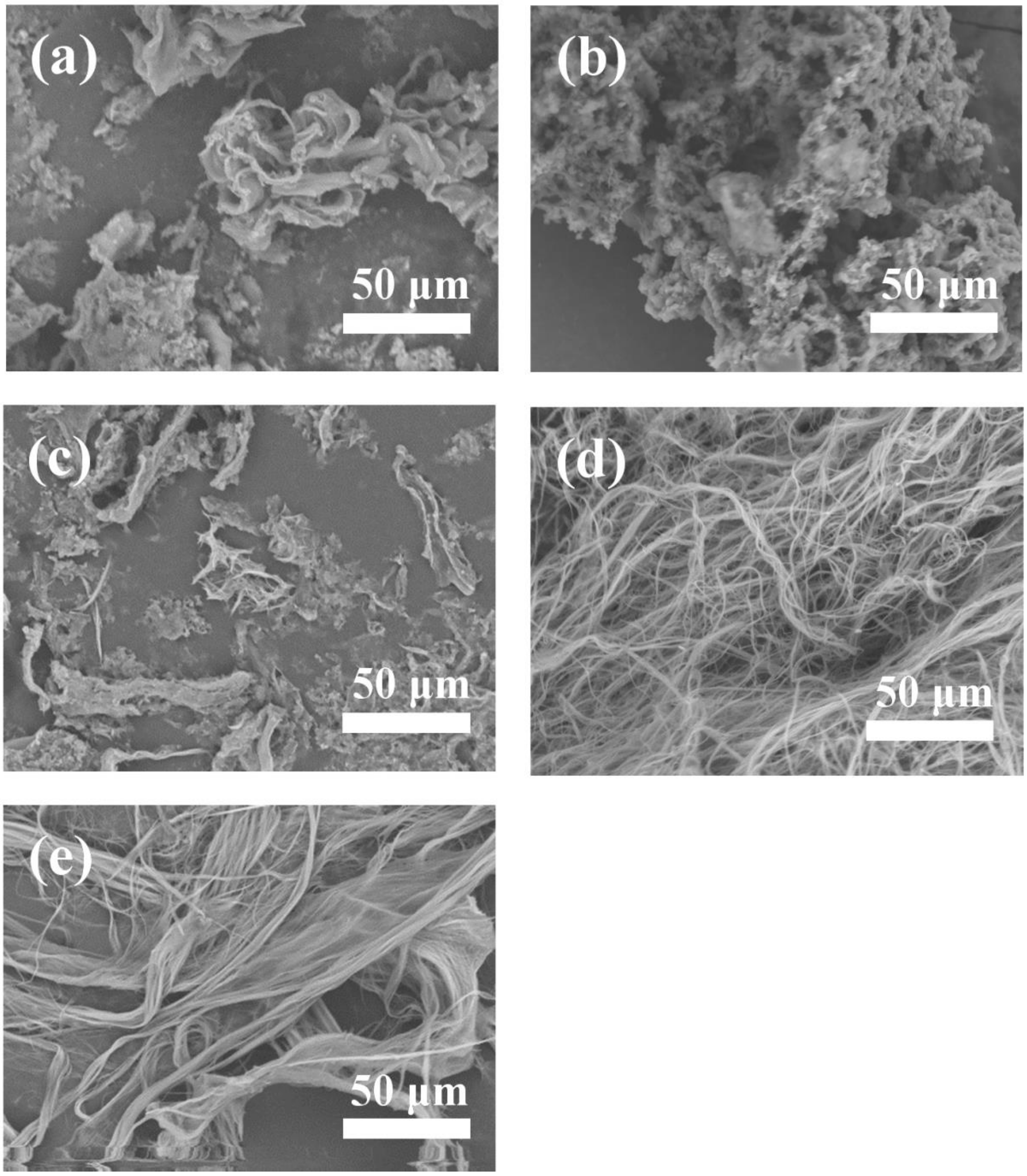

Figure S1. SEM images of the products prepared using ATP as a bio-phosphorus source in aqueous solution by the calcium oleate precursor microwave hydrothermal method for 60 min at different temperatures: (a) 120 ºC; (b) 140 ºC; (c) 160 ºC; (d) 180 ºC; (e) 200 ºC. The increase of microwave hydrothermal temperature is beneficial to the formation of ultralong HAP nanowires with higher aspect ratios.

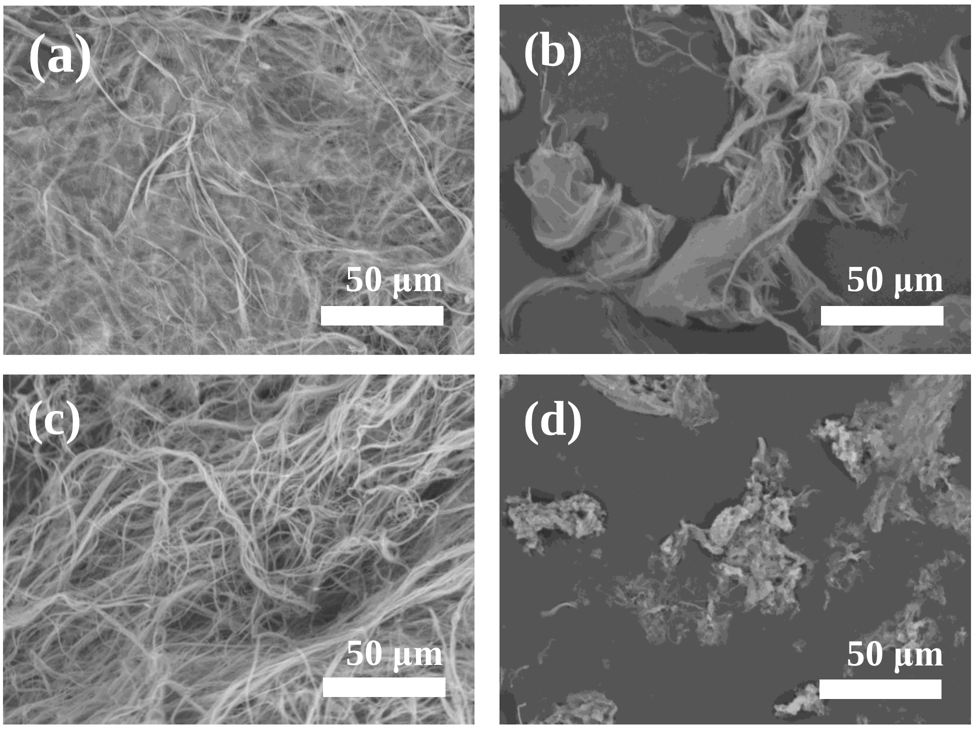

Figure S2. SEM images of the products synthesized using ATP as a bio-phosphorus source in aqueous solution by the calcium oleate precursor microwave hydrothermal method at 180 ºC for 60 min with different Ca/P molar ratios by changing the concentration of ATP: (a) 1:4; (b) 1:3; (c) 1:2; (d) 2:3. For the synthesis of ultralong HAP nanowires by the calcium oleate precursor microwave hydrothermal method at 180 ºC for 60 min, the ideal Ca/P molar ratio is 1:2.

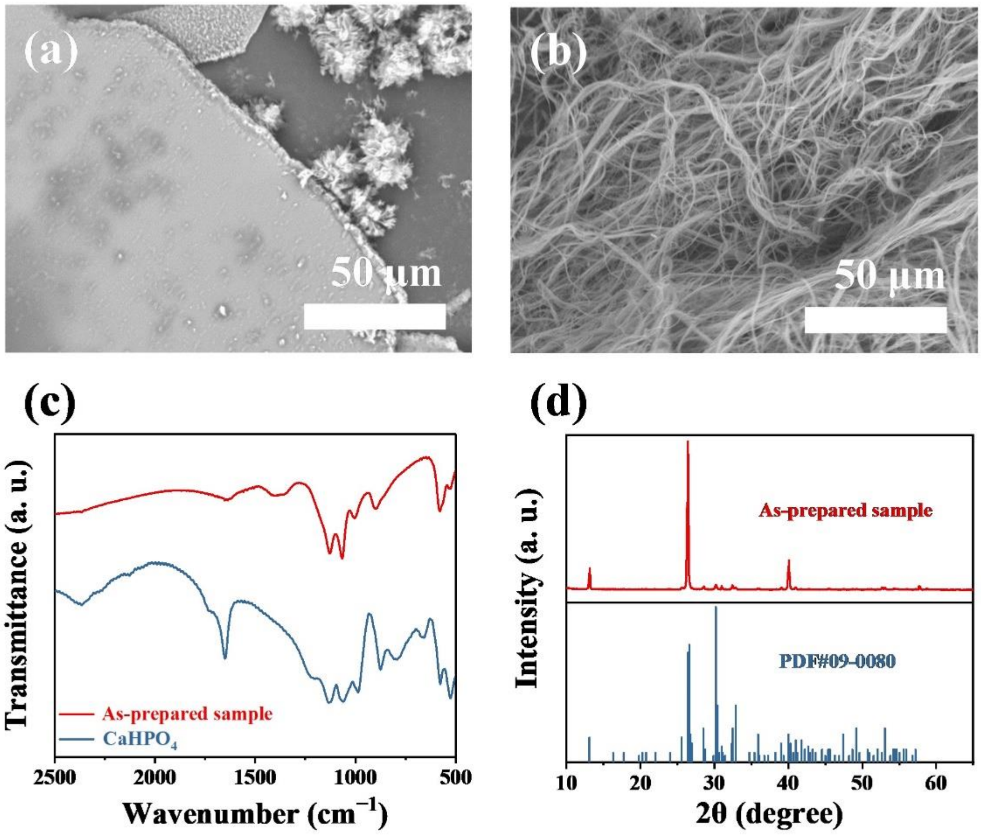

Figure S3. (a, b) SEM images of the products prepared using an aqueous solution containing $CaCl_2$ and ATP by the microwave-assisted hydrothermal method at 180 ºC for 60 min at a Ca/P molar ratio of 0.5 using different amounts of sodium oleate: (a) 0 mmol; (b) 8.65 mmol. (c) FTIR spectrum of the product prepared using an aqueous solution containing $CaCl_2$ and ATP by the microwave-assisted hydrothermal method at 180 ºC for 60 min at a Ca/P molar ratio of 0.5 in the absence of sodium oleate; in addition, FTIR spectrum of $CaHPO_4$ is presented for comparison. (d) XRD pattern of the product prepared using an aqueous solution containing $CaCl_2$ and ATP by the microwave-assisted hydrothermal method at 180 ºC for 60 min at a Ca/P molar ratio of 0.5 in the absence of sodium oleate. The presence of sodium oleate is required for the synthesis of ultralong HAP nanowires, otherwise, the product is calcium hydrogen phosphate rather than HAP.

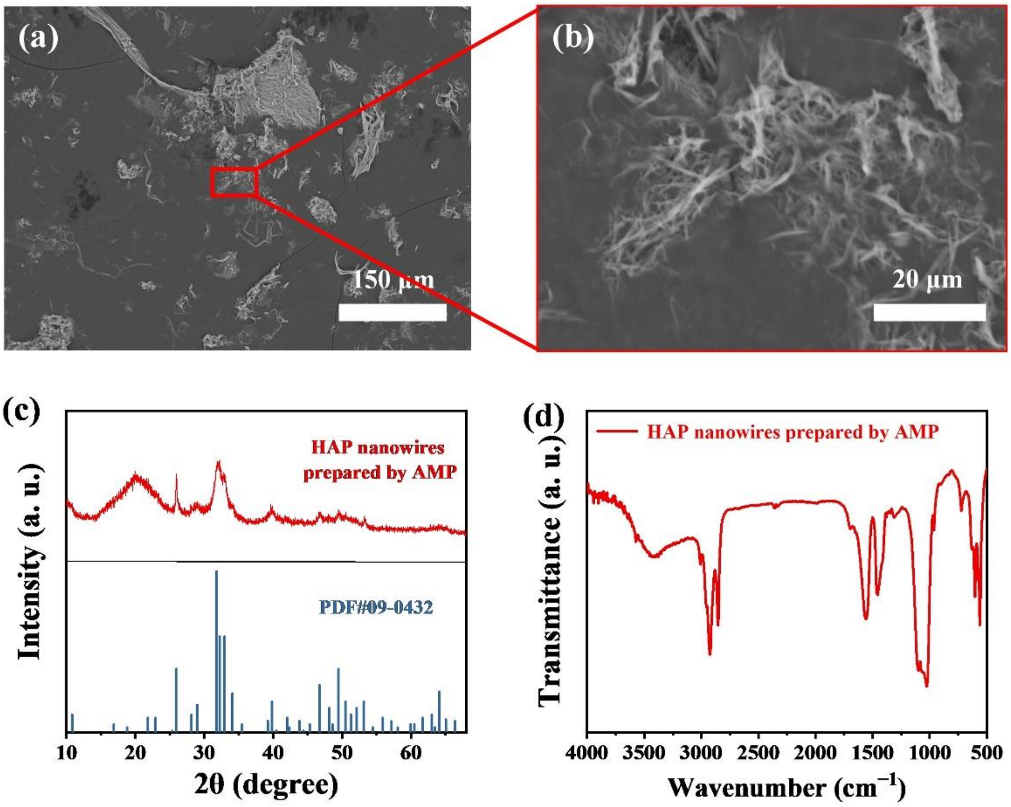

Figure S4. Characterization of the product synthesized using adenosine 5’-monophosphate (AMP) as a bio-phosphorus source in aqueous solution by the calcium oleate precursor microwave hydrothermal method at 180 ºC for 60 min. (a, b) SEM images. (c) XRD pattern. (d) FTIR spectrum. The experimental results indicate that HAP nanowires can be synthesized by the calcium oleate precursor microwave hydrothermal method using AMP as a bio-phosphorus source in aqueous solution.

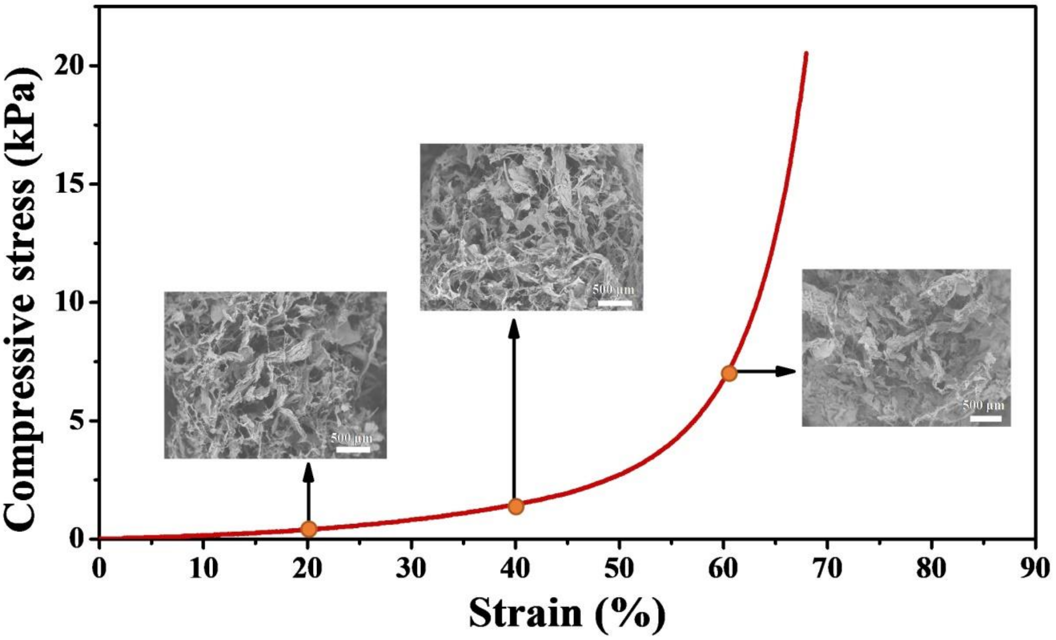

Figure S5. Compressive stress-strain curve of the ultralong HAP nanowire/CS porous scaffold, the insets are the SEM images of the ultralong HAP nanowire/CS porous scaffold after the compressive test at a strain of 20%, 40% and 60%, respectively. At a lower strain, the ultralong HAP nanowire/CS scaffold can recover to its original state after compression and deformation, exhibiting the elastic properties. When the strain increases to 40 %, the microstructure of the ultralong HAP nanowire/CS scaffold starts to suffer from serious damage, and it is not recoverable after the compression and deformation. When the strain further increases to 60 %, the pores of the ultralong HAP nanowire/CS scaffold collapse, and the deformation is completely unrecoverable after the compression test.

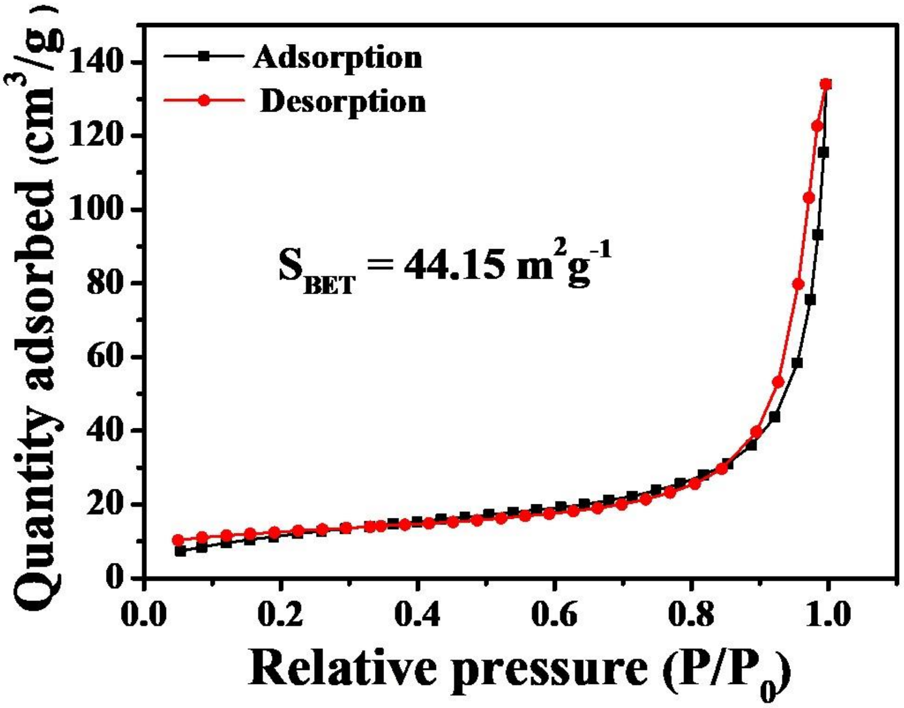

Figure S6. Nitrogen adsorption-desorption isotherms of the as-prepared ultralong HAP nanowires synthesized using ATP as a bio-phosphorus source in aqueous solution by the calcium oleate precursor microwave hydrothermal method at 180 ºC for 60 min. The specific surface area of the as-prepared ultralong HAP nanowires is measured to be 44.15 m^2 g^{-1}.
